# Associations of Content and Context of Communication with Prostate-Specific Antigen Testing

**DOI:** 10.3390/ijerph20095721

**Published:** 2023-05-04

**Authors:** Nicholas A. Alford, Manasicha Wongpaiboon, John S. Luque, Cynthia M. Harris, Rima H. Tawk

**Affiliations:** 1 Institute of Public Health, College of Pharmacy and Pharmaceutical Sciences, Florida A&M University, Tallahassee, FL 32307, USA; 2 Florida State University College of Medicine, Tallahassee, FL 32304, USA

**Keywords:** prostate cancer screening guidelines, prostate-specific antigen test, communication, shared decision-making, racial disparities

## Abstract

There is limited research about the content and context of communication on prostate-specific antigen (PSA) testing among men in the state of Florida. The purpose of this study is to understand how the content communication (discussion of advantages and disadvantages of PSA testing between provider and patient; provider recommendations of PSA testing) and the context of communication (continuity of care denoted by the presence of a personal doctor) influence PSA testing. Data were drawn from the Florida Behavioral Risk Factor Surveillance System. Receipt of PSA testing was the primary outcome. Multiple logistic regression analyses were used to adjust for sociodemographic, clinical, healthcare access, and lifestyle characteristics when associating the content and context of communication with PSA testing. Discussions were classified into four mutually exclusive categories: discussions of advantages and disadvantages, only advantages, only disadvantages, and no discussion. The most significant predictors for PSA testing included physician recommendation, discussions including advantages, older age, non-smoking, and having a personal doctor. Individualized PSA screening may be a pathway to reducing racial disparities in screening for prostate cancer (PCa) and, by extension, lower incidence and mortality rates. Developing a bill to create an Office of Men’s Health at Health & Human Services is recommended.

## 1. Introduction

In asymptomatic populations, screening remains the most common method for early detection of disease. Consensus regarding routine prostate cancer (PCa) screening with prostate-specific antigen (PSA) testing continues to be debated [1]. Two large, randomized trials, the European Randomized Study of Screening for Prostate Cancer (ERSPC) and the U.S. Prostate, Lung, Colorectal, and Ovarian (PLCO) study, have examined the relationship between screening and survival [2]. The ERSPC clinical trial exhibited a 20% relative reduction mortality in favor of screening after 11 years of follow-up but did not take into account any race-specific subgroup information [3]. In contrast, the PLCO trial reported no significant differences in PCa-specific mortality between the screening and the control arms after 13 years of follow-up, with 4% African Americans in the sample [4]. 

These two trials also revealed that PSA-based screening could produce false positive results and subsequently lead to a substantial overdiagnosis of PCa [5]. Men who have a false positive test result are more likely to undergo additional testing including one or more prostate needle biopsies compared with their negative test counterparts [5]. Following these diagnostic evaluations that lead to the identification of PCa, there remains the risk that many cases might be localized and slow-growing, and may fail to become clinically relevant [6]. In addition to the negative psychological effects, nearly 90% of PSA-diagnosed PCa cases subsequently undergo early treatment with radiation, androgen deprivation therapy, and surgery. This can result in erectile dysfunction, urinary incontinence, gynecomastia, bowel dysfunction, and hot flashes that consequently impede a patient’s quality of life [7]. Due to the potential harms and uncertainty of PSA testing in mortality reduction, organizations such as the United States Preventive Services Task Force (USPSTF), the American Urological Association (AUA), and the American College of Physicians have updated their recommendations to strongly emphasize the importance and requirement of shared decision making before ordering the test, so men have the opportunity to include their values and preferences when making the decision to be screened [8,9]. Due to the absence of unanimous guidelines across medical organizations and agencies, a balanced discussion should take place between selected patients and providers about the advantages and disadvantages of the PSA test and the scientific uncertainty of its effectiveness in reducing mortality. The extent to which men have been fully informed or involved in decisions about PSA screening and the psychosocial factors that influence their decision-making processes have not been clearly elucidated [8].

The American Cancer Society (ACS) and the AUA base their recommendations on age and life expectancy. ACS recommends discussions starting at age 50 years for men who are at average risk, age 45 years for men who are at high risk (African American men and men who have a first-degree relative, including father, brother, or son who were diagnosed at an early age), and 40 years for men at an even higher risk with more than one first-degree relative with prostate cancer at an early age [10]. Likewise, the AUA emphasizes individualized and shared decision making with men aged 50–69 and recommends against screening men 70 years old or older or any male with less than a 10–15-year life expectancy [11]. In contrast, the 2018 USPSTF guidelines categorized routine PSA screening for men ages 70 years or older as “Grade D”, discouraging the use of such screening. Cutoffs of 55 to 69 years of age are categorized as “Grade C” for periodic PSA-based screening according to individual values and preferences [12]. It is should be noted that the USPSTF recommendation of PSA screening as “Grade D” was made without sufficient data to assess its impact on different racial groups including African Americans and others [2]. In the 2018 recommendation, USPSTF emphasized that clinicians needed to consider the weighing of benefits and harms in consideration of family history, race/ethnicity, and comorbid medical conditions [13,14]. Some influential US guideline panels have expressed concerns that recent restrictive guidelines on testing have led to higher morbidity and mortality from prostate cancer and an increased incidence of metastatic prostate cancer [15].

Although discussions about benefits and risks of PSA testing are an essential element of patient-centered care and can be linked with receipt of testing, little is known about how provider recommendations and the presence of a personal doctor influence testing in the context of such discussions in Florida. Furthermore, no other studies have examined the impact of the content and context of communication between patients and providers on PSA testing in Florida. The purpose of this study is to understand how the content of communication (discussion of advantages and disadvantages of PSA testing between provider and patient; provider recommendations of PSA testing) and the context of communication (continuity of care denoted by the presence of a personal doctor) influence routine PSA testing after controlling for sociodemographic, healthcare access, and lifestyle characteristics in Florida.

## 2. Methods

### 2.1. Data Source 

The Florida Behavioral Risk Factor Surveillance System (BRFSS) survey is jointly developed and coordinated by U.S. Centers for Disease Control and Prevention (CDC) and the Florida Department of Health. The BRFSS survey is the nation’s largest telephone surveillance system covering U.S. states and territories. It targets individuals aged 18 and older and collects data on their health-related risk behaviors, chronic health conditions, and preventive health practices [16]. It can provide statewide representative estimates via iterative proportional fitting weighting. This methodology incorporates both landline and cellular telephone survey data, reduces nonresponse bias, and reduces error within estimates. Patients are weighted by age, sex, race and/or ethnicity, educational attainment, marital status, property ownership, and telephone ownership.

We used data from the 2016 Florida BRFSS to understand better the effect of communication (content and context) on the likelihood of undergoing PSA screening. 

### 2.2. Variables

The primary outcome measure was receipt of a self-reported PSA screening test which was assessed with the question: “Have you EVER HAD a PSA test?” The response options were “yes” or “no”. One of the main independent variables was patient–provider communication. Discussion of advantages was assessed with the question: “A Prostate-Specific Antigen test, also called a PSA test, is a blood test used to check men for prostate cancer. Has a doctor, nurse, or other health professional EVER talked with you about the advantages of the PSA test?” Discussion of disadvantages was assessed with the question: “Has a doctor, nurse, or other health professional EVER talked with you about the disadvantages of the PSA test?” The response options for both questions were “yes” or “no”. Consistent with a prior approach to defining of risk/benefit discussion, a 4-level variable was generated using the advantage and disadvantage discussion questions, resulting in 4 mutually exclusive categories: discussions of advantages and disadvantages, advantages only, disadvantages only, and none [9]. Provider recommendation of PSA test was assessed by asking: “Has a doctor, nurse, or other health professional EVER recommended that you have a PSA test?” Having a personal doctor was assessed with the question: “Do you have one person you think of as your personal doctor or health care provider?” The response options were “yes” (one or more than one) or “no”. 

Sociodemographic variables included age, race/ethnicity, marital status, and education. Clinical factors (comorbidity and self-rated health), access to care factors (health insurance, regular healthcare provider, delayed care due to cost), and lifestyle factors (smoking, alcohol use, and physical activity) served as other covariates of interest. Age was classified into 6 categories: “40 to 44”, “45 to 49”, “50 to 54”, “55 to 59”, “60 to 64”, and “65 to 69”. Race/ethnicity was categorized into 6 groups: “Non-Hispanic White”, “Non-Hispanic Black”, “Non-Hispanic Asian”, “Non-Hispanic American Indian/Alaskan Native”, “Hispanic”, and “Non-Hispanic other race”. Marital status was categorized into “married”, “single” (never married, member of an unmarried couple), and “other”; “other” included divorced, widowed, and separated. Education was classified into 3 categories: “less than high school”, “high school graduate”, and “college” (some college or college graduate). Comorbidities included coronary heart disease, myocardial infarction, stroke, asthma, chronic obstructive pulmonary disease, arthritis, depression, kidney disease, and diabetes. The response options were “1 disease”, “2 diseases”, “3 or more diseases”, and “none”. Self-rated health was assessed with the question: “Would you say that in general your health is:” The response options were divided into 2 categories: “fair or poor health” and “good or better health”. Healthcare access coverage was assessed with the question: “Do you have any kind of health care coverage, including health insurance, prepaid plans such as HMOs, or government plans such as Medicare, or Indian Health Service?” Medical cost concern was assessed with the question “Was there a time in the past 12 months when you needed to see a doctor but could not because of cost?” Preventive behaviors included smoking status, heavy alcohol consumption, and physical activity. Smoking status was categorized into “Everyday smoker”, “Someday smoker”, “Former smoker”, “Non-smoker”. Heavy alcohol consumption was categorized into heavy drinkers (adult men having more than 14 drinks per week), and non-heavy drinkers (14 or fewer drinks per week). Leisure-time physical activity was assessed with the question: “During the past month, other than your regular job, did you participate in any physical activities or exercises such as running, calisthenics, golf, gardening, or walking for exercise?” All responses to the risk factors listed above were dichotomized.

### 2.3. Data Analysis

The screening population was limited to men aged 40–69 years old with no history of prostate cancer. Men who reported other race such as “Non-Hispanic Asian”, “Non-Hispanic American Indian/Alaskan Natives”, “Non-Hispanic other race” were excluded because of their limited numbers. Analyses were performed using PROC SURVEYFREQ and PROC SURVEYLOGISTIC functions for complex sampling designs in SAS Version 9.4 (SAS Institute Inc., Cary, NC, USA). The odds of PSA testing receipt associated with risk/benefit discussions, provider recommendation, and presence of a personal doctor were estimated with weighted logistic regression after accounting for sociodemographic, clinical, access-to-care, and lifestyle characteristics. Covariates were selected for the multivariable models based on the literature review. We chose variables [9,17] that have been identified in the literature and added additional variables (comorbidities, and alcohol use) to make our study more comprehensive. The data were weighted to adjust for varying probabilities of selection and non-response. Adjusted odds ratios (AORs) and 95% confidence intervals (CIs) were reported. 

## 3. Results

Our sample consisted of a weighted total of 2,737,491 (*n* = 5790) participants including Non-Hispanic White, Non-Hispanic Black, and Hispanic men. Patients who had been told they had prostate cancer were excluded from the analysis (*n* = 338). Descriptive statistics for the study population were calculated as weighted percentages and are displayed in Table 1. The majority of our study population were reported as NH Whites (64.8%) followed by Hispanics (23.6%) and NH Blacks (11.6%). Over half of the study population were reported as being married (61.7%) and educated (having a college degree: 57.7%). In total, 35.4% of the study sample were between the age of 50–59. Most had relatively good access to healthcare with 83.9% being insured, 74% having a personal doctor for their care, and 15.4% reporting delays in receipt of care due to cost. Overall, 20.9% reported their health as fair or poor and 48.1% reported no chronic conditions. With respect to their lifestyle characteristics, 19.9% were smokers, 9.2% were heavy drinkers, and 29.2% were physically inactive. 

Table 2 lists the prevalence estimates of patient–provider discussion of the risks and benefits of PSA testing. The unadjusted association of patient–provider communication with PSA testing is also reported. Nearly 44% (43.8%) of men reported no previous physician–patient discussion of advantages or disadvantages. Over than half of the respondents (56.2%) reported some form of discussion with their providers: 29.1% of respondents reported discussion of advantages only; 26.5% of respondents reported discussion of advantages and disadvantages; 0.6% of respondents reported discussion of disadvantages only. 

According to the multivariable analysis (Table 3), men whose providers recommended testing were more likely to receive PSA testing (AOR = 14.90; *p* < 0.0001) compared with men whose providers had never recommended PSA testing. With regard to discussion of risks and benefits of PSA testing, men who discussed only advantages or discussed both were more likely to undergo PSA testing (AOR = 8.39; *p* < 0.0001 and AOR = 6.05; *p* < 0.0001, respectively) compared with those who had no discussions. Men who only discussed disadvantages with providers did not differ from those who had no discussions (*p* = 0.1252). Men who had a personal doctor were 88% more likely to undergo testing compared with those who did not have a personal doctor. The odds of PSA testing varied by age groups compared with the youngest category: the 40–44 age group. The highest likelihood of PSA testing was reported for the 60–64 age group (AOR = 5.91) while the 45–49 age group had the lowest likelihood of PSA testing (AOR = 1.58). Race failed to reach statistical significance in PSA testing in the adjusted model. However, in the unadjusted model, race was significant, with the minority group non-Hispanic Blacks and Hispanics having a lower likelihood of PSA testing (OR = 0.65 (0.46–0.92); *p* = 0.0148 and OR = 0.67 (0.51–0.91), respectively; *p* = 0.0088) compared with Non-Hispanic Whites (results not shown here). Compared with uninsured men, insured men were about 79% more likely to undergo testing. With respect to everyday smoking, non-smokers had the highest likelihood of PSA testing (AOR = 2.29) followed by former smokers (AOR = 2.17). Marital status, education, self-rated health, comorbidities, medical cost concern, occasional smoking, heavy alcohol use, and physical activity were not significant predictors for PSA testing. 

The multivariable logistic regression model adjusted for age, race/ethnicity, marital status, education, insurance status, having a regular provider, self-rated health, delayed care due to cost, smoking status, heavy alcohol consumption, physical activity, patterns of patient–provider discussions, provider recommendation of PSA test, and comorbidities. 

## 4. Discussion

In this study, we tested the association between content and context of communication and receipt of PSA screening. Our findings suggest that men who engaged in conversations with their healthcare provider and were given recommendations had a greater likelihood of being in receipt of PSA than those who did not engage in those conversations. In a 2015 study, it was shown that discussions of advantages of PSA testing alone or discussions of both advantages and disadvantages of PSA testing were associated with a higher prevalence of receipt of PSA testing [9]. Those findings are consistent with this study’s findings, except for discussion of disadvantages. Another study reported no significant differences between men who only discussed disadvantages with their providers and men who had no discussions [17]. This result was in agreement with our findings. Potential explanations for the association between discussing both advantages and disadvantages and a higher likelihood of undergoing PSA screening might be that: (1) healthcare providers emphasized benefits more frequently than harms or gave greater weight to benefits; and (2) healthcare providers and patients had a balanced shared decision-making discussion of risks and benefits [9] and ultimately, undergoing PSA testing was the patient’s preference; or (3) patients had a preconceived notion of wanting to undergo screening and had already made that decision prior to the discussion [9]. 

Generally, physician recommendation has been shown to be strongly associated with the decision to have a PSA test. Our results showed that physician recommendation was the most significant predictor of screening, and these results agreed with those of another study [17]. 

Shared decision making between patients and providers is a recurrent theme and is embedded in the PSA guidelines of several health organizations outside the United States, including the International Society of Urological Pathology, the Urological Society of Australia and New Zealand, United Kingdom National Institute for Health and Care Excellence, and the European Association of Urology [18]. A longitudinal study incorporated 13 Dutch clinical facilities to evaluate the accuracy of patients’ perceptions of risks associated with localized PCa treatments (radiotherapy, active surveillance, radical prostatectomy) [19]. Results from that study found that two-thirds of the patients poorly understood the risks associated with each treatment. That study led Hochstenbach et al. to seek improvements in patient PCa education by improving shared decision making through the use of web-based patient-decision aids [20]. Those aids included a representation of generalized and personalized risks of side effects.

We hypothesized that race/ethnicity would be a significant predictor in this study. African Americans are disproportionately affected by PC and are more likely to harbor tumor (1.74 higher PC incidence) [2] or succumb (2.4-fold increase in mortality) [21] to these than their White counterparts. Racial disparities in PC incidence and mortality are multifactorial and complex and may be explained by factors that are biological, social such as racism and discrimination [22], environmental, or healthcare-related [23] including prevention, early detection of cancer, and evidence-based treatment [24]. Some studies have shown that many African American men do not communicate well with their regular healthcare providers [25]. While shared decision making is recommended prior to PSA screening, several studies highlight that African American men may not be making informed decisions about PSA screening due to patients having low health literacy, knowledge about the test, and past history of receiving PSA [6,26,27]. Moreover, healthcare providers may not be offering sufficient up-to-date information or may not be asking patients about their preferences, thereby hindering the shared decision-making process [28].

When compared with other countries with diverse racial and ethnic populations, such as Canada and the UK, conversations in the USA regarding PSA screening are solely based on the patient’s choice. Currently, no prostate cancer screening program exists in the UK, and having a policy for routine screening of men aged 50–74 years would cause additional costs for the healthcare system. General practitioners are not encouraged to proactively raise the issue of PSA testing. Asymptomatic men over the age of 50 who wish to have a test can do so after careful consideration [29]. There still remains controversy about PSA screening. The European Randomized Study of Screening for Prostate Cancer (ERSPC) suggests that screening may reduce the long-term risk of prostate-cancer-specific mortality. This suggestion is why many European countries are against systematic PSA-based screening for prostate cancer [30], and coincides with the Canadian Task Force on Preventive Health Care PSA guidance [31]. Balanced conversations regarding screening remain problematic due to uncertainty in the evidence of its benefits [32]. 

The Institute of Medicine has shown that minorities are less likely to undergo recommended cancer screening and that adverse outcomes are more prevalent in minority populations [24]. Other studies have examined the combined effects of race and income. Factors and barriers that impact patients’ decisions to undergo PCa screening include input from family and peers, lack of transportation, financial resources, or mistrust of the healthcare system [33]. Even when access-related factors are controlled, according to the 2002 Institute of Medicine report Unequal Treatment: Confronting Racial and Ethnic Disparities in Health Care, there is evidence that racial and ethnic minorities tend to receive lower-quality healthcare than Whites [34]. Marked differences in risk factors for cancer, such as high-calorie fatty diets and smoking, are observed among different Hispanic populations in the US. For example, Cubans and Puerto Ricans are characterized by higher PCa risk, possibly associated with their longer average time spent in the USA, compared with other Hispanic immigrants [35]. Dominicans, a population with African ancestry, have double the PCa mortality compared with Non-Hispanic Whites and have low rates of PCa screening [35]. For African American men, knowledge, attitudes and values, fear, attributes of masculinity, and communication have all been previously identified as barriers to health screening [10,33].

Other independent predictors of PSA screening include education, income, marriage status, preventive behaviors, and access to care [36]. Drazer et al. found higher PSA screening rates in men who were married or living with a partner, and those who had higher levels of education, family history of cancer, and Medicare coverage, had seen a general practitioner in the prior year, participated in moderate physical activity, were current or former alcohol drinkers, and those who took vitamins [36]. Men in these groups are often more health conscious or have more social support than those who do not adhere to medical advice. We found that some of these predictors such as marital status, education, heavy alcohol use, and physical activity were not identified as predictors of PSA screening among men in Florida. 

Similarly, industrialized European countries also lack universal PCa testing guidelines. There, screening is performed opportunistically, that is, based on the individual’s decision rather than in an “organized” fashion [37]. In the European Union, the screening of several other cancers such as breast, cervical, and colorectal cancer has been implemented through “organized” programs where screening guidelines and policies are explicitly structured [38]. However, the lack of programs for PCa screening has disproportionately affected certain sociodemographic populations [39]. Using data from the Finnish Randomized Study of Screening for Prostate Cancer database, a subset of the ERSPC, Kilpeläinen et al. found that higher educational level, higher income, and home ownership were associated with increased levels of participation in PCa screening and incidence among Finnish residents [40]. They attributed the increased incidence of PCa among high socioeconomic status (SES) compared with their low SES counterparts to the insufficient number of low SES patients who seek medical care and PCa screening [40].

Currently, there is a push to improve community efforts and clinical management to promote prostate health and PSA screening in Florida. For example, there have been advocates promoting the establishment of an Office of Men’s Health in the Department of Health. We encourage the development of a bill at the state government level to create an Office of Men’s Health at Health & Human Services. Only 14 out of 50 states have an office or coordinator of men’s health. Health organizations and community groups should raise further awareness about men’s health and prostate cancer. Educating providers and patients alike about screening guidelines, benefits, and harms is helpful for developing preventative care plans. In partnership with their providers, patients are encouraged to communicate their preferences and help select the course of action that matches their values and preferences [41]. Individualized PSA screening may be a pathway to reducing racial disparities in screening for PCa and, by extension, lower incidence and mortality rates. A personalized medicine approach including the underlying individual risk factors and genomic traits is warranted in addressing disparities in incidence and mortality of PCa, rather than a focus only on racial and ethnic groups which has shown limited efficacy in PCa prevention and treatment [23]. 

## 5. Limitations

Our study is not devoid of limitations. Data may not be nationally representative, because our dataset is limited to respondents residing in Florida. Another limitation is that the dataset represents a cross-sectional study and thus cannot determine causality. Due to the self-reported survey data, this study is also predisposed to recall bias and non-response bias. PSA testing may be underreported, such as in the event that providers ordered the test without informing the patient (i.e., as a part of routine wellness examination). The level of underreporting or overreporting of health-related behaviors also cannot be determined. Responses to questions about screening discussions were framed as yes or no, and the specific content of the discussions and whether these discussions were balanced could not be captured by the dataset. Patients’ knowledge, values, and preferences relating to PSA testing were not collected. Interpretations of the survey’s discussion questions may have differed among patients according to their levels of health literacy. 

## 6. Conclusions

Men who engaged in conversations with their physician, who were given recommendations for PSA testing by their providers, and who benefitted from the continuity of care denoted by the presence of a personal doctor had a greater likelihood of PSA receipt than those who did not experience these elements of patient-centered care. To our knowledge, little to no other research has examined the interaction between patient–provider communication and PSA adherence in Florida after adjusting for sociodemographic, clinical, and lifestyle characteristics and access to care. Our study showed that in the state of Florida, race was not a predictor for engaging in prostate cancer-related communication. The most significant predictors included physician recommendation, discussions including advantages, older age (60–64), access-related factors (having a personal doctor, insurance). Other predictors with statistical significance on PSA screening included preventive behaviors such as being a non-smoker or a former smoker. 

## Figures and Tables

**Table 1:** Characteristics of Weighted Study Population (N * = 2,737,491), 2016 Florida Behavioral Risk Factor Surveillance System.

Variable		N	Percent
Age	40–49	889,808	32.5
	50–59	968,547	35.4
	60–69	879,136	32.1
Race/Ethnicity	Non-Hispanic Whites	1,773,866	64.8
	Non-Hispanic Blacks	318,502	11.6
	Hispanics	645,123	23.6
Marital Status	Married	1,689,190	61.7
	Single	450,234	16.4
	Other	598,067	21.9
Education	<High school	429,058	15.7
	High school	728,816	26.6
	College	1,579,617	57.7
Insurance Status	Uninsured	439,623	16.1
Presence of Disease	0	1,317,514	48.1
	1–2	1,062,244	38.8
	3+	357,733	13.1
Have A Personal Doctor	No	711,817	26.0
General Health	Fair or Poor	572,272	20.9
Medical Cost Concern	Yes	420,417	15.4
Heavy Alcohol Use	Yes	250,662	9.2
Physical Inactivity	Yes	798,112	29.2
Smoking	Everyday smoker	357,842	13.1
	Someday smoker	187,593	6.8
	Former smoker	931,433	34.0
	Non-smoker	1,260,623	46.1

* Total is estimated using sampling weights. Unweighted total is 5790.

**Table 2:** Prevalence Estimates and Unadjusted ORs of Patient–Provider Communication for PSA Testing.

Variable		N	Percent	Odds Radio	LCL	UCL	*p*-Value
Patient–provider communication	None	1,177,975	43.8	1.0			
	Advantages only	779,508	29.1	30.12	21.23	42.73	<0.0001
	Disadvantages only	16,717	0.6	7.89	2.70	23.02	0.0002
	Both	711,980	26.5	26.85	18.70	38.54	<0.0001

Abbreviations: LCL: lower confidence interval; UCL: upper confidence interval.

**Table 3:** Adjusted Associations of Patient–Provider Communication with PSA Testing, 2016 Florida Behavioral Risk Factor Surveillance System.

	Adjusted Odds Ratios	LCL	UCL	*p*-Value
Pre-screening patient–provider discussions				
Advantages only	8.39	5.57	12.64	<0.0001
Disadvantages only	2.35	0.79	6.99	0.1252
Both	6.05	4.02	9.12	<0.0001
None	1.0			
Provider recommended PSA				
Yes	14.90	10.60	20.93	<0.0001
No	1.0			
Personal doctor				
Yes (one or more than one)	1.88	1.19	2.97	0.0068
No	1.0			
Medical cost concern				
Yes	1.32	0.74	2.36	0.3472
No	1.0			
Age				
40–44	1.0			
45–49	1.58	0.87	2.89	0.1344
50–54	3.07	1.64	5.73	0.0004
55–59	2.64	1.49	4.69	0.0009
60–64	5.91	3.16	11.06	<0.0001
65–69	4.39	2.25	8.55	<0.0001
Race/Ethnicity				
Non-Hispanic White	1.0			
Non-Hispanic Black	0.93	0.57	1.51	0.7790
Hispanic	1.45	0.85	2.47	0.1673
Marital status				
Single	1.0			
Married	1.39	0.85	2.29	0.1844
Other	0.93	0.53	1.62	0.7884
Education				
Less than high school	1.0			
High school	1.52	0.76	3.05	0.2325
College	1.53	0.80	2.96	0.2004
Self-rated health				
Fair or poor	1.0			
Good or better health	0.85	0.53	1.36	0.4904
Health insurance				
Yes	1.78	1.02	3.12	0.0407
No	1.0			
Comorbidities				
One disease	1.03	0.69	1.53	0.8925
Two diseases	0.99	0.60	1.64	0.9680
Three or more diseases	1.60	0.89	2.87	0.1124
None	1.0			
Smoking status				
Everyday smoker	1.0			
Someday smoker	1.70	0.68	4.25	0.2588
Former smoker	2.17	1.17	4.00	0.0130
Non-smoker	2.29	1.24	4.22	0.0078
Heavy alcohol use				
Yes	0.96	0.58	1.58	0.8631
No	1.0			
Physical activity				
Yes	1.66	1.11	2.49	0.0139
No	1.0			

Abbreviations: LCL: lower confidence interval; UCL: upper confidence interval.

## Data Availability

All data generated from this research are presented in this manuscript.

## References

[1] Fleshner K., Carlsson S.V., Roobol M.J. (2017). The Effect of the USPSTF PSA Screening Recommendation on Prostate Cancer Incidence Patterns in the USA. Nat. Rev. Urol..

[2] Arace J., Flores V., Monaghan T., Robins D., Karanikolas N., Winer A., Weiss J. (2020). Rates of Clinically Significant Prostate Cancer in African Americans Increased Significantly Following the 2012 US Preventative Services Task Force Recommendation against Prostate Specific Antigen Screening: A Single Institution Retrospective Study. Int. J. Clin. Pract..

[3] Schröder F.H., Hugosson J., Roobol M.J., Tammela T.L.J., Ciatto S., Nelen V., Kwiatkowski M., Lujan M., Lilja H., Zappa M. (2012). ERSPC Investigators. Prostate-Cancer Mortality at 11 Years of Follow-Up. N. Engl. J. Med..

[4] Andriole G.L., Crawford E.D., Grubb R.L., Buys S.S., Chia D., Church T.R., Fouad M.N., Gelmann E.P., Kvale P.A., Reding D.J. (2009). Mortality Results from a Randomized Prostate-Cancer Screening Trial. N. Engl. J. Med..

[5] Moyer V.A., U.S. Preventive Services Task Force (2012). Screening for Prostate Cancer: U.S. Preventive Services Task Force Recommendation Statement. Ann. Intern. Med..

[6] Leyva B., Persoskie A., Ottenbacher A., Hamilton J.G., Allen J.D., Kobrin S.C., Taplin S.H. (2016). Do Men Receive Information Required for Shared Decision Making About PSA Testing? Results from a National Survey. J. Cancer Educ..

[7] Kearns J.T., Holt S.K., Wright J.L., Lin D.W., Lange P.H., Gore J.L. (2018). PSA Screening, Prostate Biopsy, and Treatment of Prostate Cancer in the Years Surrounding the USPSTF Recommendation against Prostate Cancer Screening. Cancer.

[8] Allen J.D., Othus M.K., Hart A., Mohllajee A.P., Li Y., Bowen D. (2011). Do men make informed decisions about prostate cancer screening? Baseline results from the “take the wheel” trial. Med. Decis. Making.

[9] Li J., Zhao G., Hall I.J. (2015). Pre-Screening Discussions and Prostate-Specific Antigen Testing for Prostate Cancer Screening. Am. J. Prev. Med..

[10] Cooper D.L., Rollins L., Slocumb T., Rivers B.M. (2019). Are Men Making Informed Decisions According to the Prostate-Specific Antigen Test Guidelines? Analysis of the 2015 Behavioral Risk Factor Surveillance System. Am. J. Mens Health.

[11] Carter H.B., Albertsen P.C., Barry M.J., Etzioni R., Freedland S.J., Greene K.L., Holmberg L., Kantoff P., Konety B.R., Murad M.H. (2013). Early Detection of Prostate Cancer: AUA Guideline. J. Urol..

[12] Burgess L., Aldrighetti C.M., Ghosh A., Niemierko A., Chino F., Huynh M.J., Efstathiou J.A., Kamran S.C. (2022). Association of the USPSTF Grade D Recommendation Against Prostate-Specific Antigen Screening with Prostate Cancer–Specific Mortality. JAMA Network Open.

[13] Grossman D.C., Curry S.J., Owens D.K., Bibbins-Domingo K., Caughey A.B., Davidson K.W., Doubeni C.A., Ebell M., Epling J.W., U.S. Preventive Services Task Force (2018). Screening for Prostate Cancer: US Preventive Services Task Force Recommendation Statement. JAMA.

[14] Kensler K.H., Pernar C.H., Mahal B.A., Nguyen P.L., Trinh Q.-D., Kibel A.S., Rebbeck T.R. (2021). Racial and Ethnic Variation in PSA Testing and Prostate Cancer Incidence Following the 2012 USPSTF Recommendation. J. Natl. Cancer Inst..

[15] Gulati R., Tsodikov A., Etzioni R., Hunter-Merrill R.A., Gore J.L., Mariotto A.B., Cooperberg M.R. (2014). Expected Population Impacts of Discontinued Prostate-Specific Antigen Screening. Cancer.

[16] Behavioral Risk Factor Surveillance System (BRFSS)|Florida Department of Health. https://www.floridahealth.gov/statistics-and-data/survey-data/behavioral-risk-factor-surveillance-system/index.html.

[17] Liao J.M., Ommerborn M.J., Clark C.R. (2017). Association between Features of Patient-Provider Discussions and Routine Prostate-Specific Antigen Testing. PLoS ONE.

[18] Tse T., Wu B., Vagholkar S., Willcock S. (2023). Shared Decision Making in Prostate Cancer Screening: An Update. Aust. J. Gen. Pract..

[19] Van Stam M.-A., van der Poel H.G., van der Voort van Zyp J.R.N., Tillier C.N., Horenblas S., Aaronson N.K., Ruud Bosch J.L.H. (2018). The Accuracy of Patients’ Perceptions of the Risks Associated with Localised Prostate Cancer Treatments. BJU Int..

[20] Hochstenbach L.M.J., Determann D., Fijten R.R.R., Bloemen-van Gurp E.J., Verwey R. (2023). Taking Shared Decision Making for Prostate Cancer to the next Level: Requirements for a Dutch Treatment Decision Aid with Personalized Risks on Side Effects. Internet Interv..

[21] DeSantis C.E., Siegel R.L., Sauer A.G., Miller K.D., Fedewa S.A., Alcaraz K.I., Jemal A. (2016). Cancer Statistics for African Americans, 2016: Progress and Opportunities in Reducing Racial Disparities. CA Cancer J. Clin..

[22] Crittendon D.R., LaNoue M., George B. (2022). Does Perceived Racism Affect Prostate Cancer Screening Rates and Patient-Provider Shared Discussions Among Black and White Men?. J. Health Care Poor Underserved.

[23] Rebbeck T.R. (2018). Prostate Cancer Disparities by Race and Ethnicity: From Nucleotide to Neighborhood. Cold Spring Harb. Perspect. Med..

[24] Roberts L.R., Wilson C.M., Stiel L., Casiano C.A., Montgomery S.B. (2018). Prostate Cancer Screening among High-Risk Black Men. J. Nurse Pract..

[25] Miller D.B., Markt S.C., Nguyen C.T., Coleman O.C. (2022). Prostate Cancer Screening and Young Black Men: Can Early Communication Avoid Later Health Disparities?. J. Cancer Educ..

[26] Coughlin S.S., Ayyala D.N., Luque J.S., Moore J.X. (2021). Predictors of Prostate Cancer Screening among African American Men Treated at an Academic Medical Center in the Southern United States. Curr. Cancer Rep..

[27] Luque J.S., Ross L., Gwede C.K. (2016). Prostate Cancer Education in African American Barbershops: Baseline Client Survey Results and Differences in Decisional Conflict and Stage of Decision Making. Am. J. Mens Health.

[28] Woods-Burnham L., Stiel L., Wilson C., Montgomery S., Durán A.M., Ruckle H.R., Thompson R.A., De León M., Casiano C.A. (2018). Physician Consultations, Prostate Cancer Knowledge, and PSA Screening of African American Men in the Era of Shared Decision-Making. Am. J. Mens Health.

[29] Nderitu P., Van Hemelrijck M., Ashworth M., Mathur R., Hull S., Dudek A., Chowdhury S. (2016). Prostate-specific antigen testing in inner London general practices: Are those at higher risk most likely to get tested?. BMJ Open.

[30] Tikkinen K.A., Dahm P., Lytvyn L., Heen A.F., Vernooij R.W., Siemieniuk R.A., Wheeler R., Vaughan B., Fobuzi A.C., Junod N. (2018). Prostate cancer screening with prostate-specific antigen (PSA) test: A clinical practice guideline. BMJ.

[31] Bell N., Gorber S.C., Shane A., Joffres M., Singh H., Dickinson J., Shaw E., Dunfield L., Tonelli M., Canadian Task Force on Preventive Health Care (2014). Canadian Task Force on Preventive Health Care. Recommendations on screening for prostate cancer with the prostate-specific antigen test. CMAJ.

[32] Ransohoff D.F., McNaughton M.C., Floyd J.F. (2002). Why is prostate cancer screening so common when the evidence is so uncertain? A system without negative feedback. Am. J. Med..

[33] Hewitt T., Killinger K.A., Hiller S., Boura J.A., Lutz M. (2018). Exploring Racial Differences Surrounding Prostate Cancer Screening: Beliefs and Attitudes in Community Dwelling Men Attending an Urban Men’s Health Event. Am. J. Mens Health.

[34] Wu I., Modlin C.S. (2012). Disparities in Prostate Cancer in African American Men: What Primary Care Physicians Can Do. Cleve Clin. J. Med..

[35] Pinheiro P.S., Callahan K.E., Siegel R.L., Jin H., Morris C.R., Trapido E.J., Gomez S.L. (2017). Cancer Mortality in Hispanic Ethnic Groups. Cancer Epidemiol. Biomarkers Prev..

[36] Drazer M.W., Huo D., Schonberg M.A., Razmaria A., Eggener S.E. (2011). Population-Based Patterns and Predictors of Prostate-Specific Antigen Screening Among Older Men in the United States. J. Clin. Oncol..

[37] Roobol M.J. (2018). Screening for Prostate Cancer: Are Organized Screening Programs Necessary?. Transl. Androl. Urol..

[38] Dominitz J.A., Levin T.R. (2020). What Is Organized Screening and What Is Its Value?. Gastrointest. Endosc. Clin. North Am..

[39] Alterbeck M., Järbur E., Thimansson E., Wallström J., Bengtsson J., Björk-Eriksson T., Bjartell A., Bratt O., Jiborn T., Arnsrud Godtman R. (2022). Designing and Implementing a Population-Based Organised Prostate Cancer Testing Programme. Eur. Urol. Focus.

[40] Kilpeläinen T.P., Talala K., Raitanen J., Taari K., Kujala P., Tammela T.L.J., Auvinen A. (2016). Prostate Cancer and Socioeconomic Status in the Finnish Randomized Study of Screening for Prostate Cancer. Am. J. Epidemiol..

[41] Stiggelbout A.M., Van der Weijden T., De Wit M.P.T., Frosch D., Légaré F., Montori V.M., Trevena L., Elwyn G. (2012). Shared Decision Making: Really Putting Patients at the Centre of Healthcare. BMJ.

